# Identification of Anti-staphylococcal and Anti-biofilm Compounds by Repurposing the Medicines for Malaria Venture Pathogen Box

**DOI:** 10.3389/fcimb.2018.00365

**Published:** 2018-10-23

**Authors:** Vasundhra Bhandari, Shalini Chakraborty, Umarani Brahma, Paresh Sharma

**Affiliations:** National Institute of Animal Biotechnology, Hyderabad, India

**Keywords:** anti-staphylococcal, anti-biofilm, repurposing, antimicrobial resistance, open source drug discovery

## Abstract

There has been an alarming increase in infections caused by antimicrobial-resistant pathogens. These infections are responsible for more than half a million deaths globally each year. *Staphylococcus aureus* is one of the deadliest bacterial pathogen responsible for nosocomial and community acquired infections. The open-access Pathogen Box (PBox) provides a potential platform to identify new treatment options against antibiotic-resistant bacteria by repurposing it. In this study, we have screened the PBox library comprised of ~400 compounds to identify novel anti-staphylococcal compounds. *in vitro* antimicrobial screening using *S. aureus* isolates, ATCC 29213 (methicillin-sensitive) and ATCC 700699 (methicillin-resistant) revealed 13 compounds which showed highly potent antibacterial activity against both planktonic and biofilm state. The 13 compounds were not found cytotoxic to mouse macrophage cell line, RAW264.7. Out of the 13 compounds, only MMV687251 and MMV676477 revealed structural similarity with vancomycin by comparing their atomic pair fingerprints using Tanimoto coefficient method. The structural similarities may indicate similar mode of action like vancomycin for the two compounds. Our result showed that PBox compounds offer a promising lead for the development of new anti-staphylococcal treatment options.

## Introduction

*Staphylococcus aureus* is an opportunistic pathogen, which contributes to a significant health problem in both the clinical and community settings, especially methicillin-resistant *S. aureus* (MRSA) (Diep et al., 2008). MRSA causes mild to severe infection of various body tissues, and frequently progress to life-threatening diseases (Otto, 2012). MRSA infections represent a mortality rate of 20% and are challenging to treat due to limited treatment options (Klevens et al., 2007). The World Health Organization has kept *S. aureus* under the list of high priority pathogen against which new treatments options are required (World health Organization (WHO report)., 2017). *S. aureus* is also known to cause substantial proportions of infections in patients with indwelling medical devices (catheter, implants etc.), due to its ability to form biofilms (LaPlante and Mermel, 2009). Biofilm provides bacteria with an added benefit to evade immune responses and antibiotic effect resulting in much complicated and challenging treatment.

Reports of isolates with reduced susceptibility and resistance to vancomycin, the traditional choice of treatment against MRSA has further raised the concern about the scarcity of new treatment options (Hiramatsu et al., 1997; Howden et al., 2010). Most traditional antibiotics have significantly reduced antimicrobial activities in biofilm-associated infections (LaPlante and Mermel, 2009). Therefore, we have screened the diverse library of compounds (400 drug-like compounds) assembled by Medicine for Malaria Venture (MMV) referred as Pathogen Box® (PBox), to find new treatment option against *S. aureus* (Medicines for Malaria Venture, 2011).

The PBox compounds have been categorized into pathogen-specific subsets, the majority of the compounds have activity against *Plasmodium*, followed by tuberculosis and other Kinetoplastids. Very few compounds with activity against others pathogens: Helminths, *Toxoplasma, Dengue* and *Cryptosporidium* are also present in the PBox. The PBox compounds have not been tried against the other pathogens and therefore provides researchers with an opportunity to screen and identify new leads against pathogens with limited treatment options. The PBox compounds were reported to be 5-fold less cytotoxic against the human cell line in comparison to the pathogen, which is within the acceptable levels (Medicines for Malaria Venture, 2011) (https://www.pathogenbox.org/about-pathogen-box/supporting-information/).

Thus, in the present study, we have utilized this opportunity to identify antibacterial agent by repurposing the MMV PBox against planktonic and biofilm state of *S. aureus*.

## Materials and methods

### Bacterial and cell culture

ATCC 29213 (methicillin-susceptible *S. aureus* Sensitive and CLSI recommended isolate for broth microdilution assay) and ATCC 700699 (methicillin-resistant *S. aureus* with reduced vancomycin susceptibility) were used for antimicrobial susceptibility and anti-biofilm assays. RAW 264.7 mouse macrophage cell line was used for cytotoxicity assay. In all experiments, vancomycin and oxacillin were used as internal control.

### Antimicrobial susceptibility assay

The broth microdilution assay was performed in accordance with Clinical and Laboratory Standards Institute (CLSI) with minor modification by the use of resazurin dye as described earlier (Elshikh et al., 2016; Mahato et al., 2017). Resazurin dye (blue) is a redox indicator, which changes color from blue to pink (resorufin dye) by the metabolically active cells. The assay was performed for all the PBox compounds ranging from (0 to 100 μM) provided by MMV in a 96 well plate format. After 24 h incubation of the bacterial cells with different dilutions of PBox compounds at 37°C, 30 μl of resazurin dye (0.015 %) was added to all wells, followed by 2–4 h of incubation. The lowest dilution columns with no color change were reported as the MIC value.

All assays were performed at least twice in triplicates.

### Anti-biofilm activity

The activity of PBox compounds was tested on *S. aureus* biofilms by Crystal violet (CV) assay in a 96-well plate-format (Coraça-Huber et al., 2012; Masadeh et al., 2013; Ghosh et al., 2015). An overnight cultures of *S. aureus* (ATCC 29213 and ATCC 700699) was diluted 1:200 in Tryptic Soy Broth (TSB) with 0.25 % glucose. Two hundred microliter /well of the diluted culture was dispensed into 96-well plate. The plate was incubated for 24 h at 37°C and washed thrice with PBS. Compounds ranging from 0 to 100 μM, were added to wells and the plate was further incubated for 24 h at 37°C. The wells were then washed thrice with PBS and methanol fixed for 15 min. The plate was air dried for 30 min and 0.1% CV solution was added to each well and incubated at room temperature for 20 min. After washing with distilled water, 33% acetic acid was added to each well and absorbance was taken at 590 nm using a multimode reader (Enspire, Perkin Elmer). Mean absorbance values of each sample was calculated and compared with the mean value of the control. All assays were performed at least twice in triplicates.

### Cell cytotoxicity

The cell cytotoxicity assay was performed using RAW 264.7 mouse macrophage adherent cell line as described previously with slight modifications (Hua et al., 2018). Cells at a density of 5000 cells per well were seeded in 96 well tissue culture plate and allowed to adhere in DMEM medium (10% FBS) for 24 h at 37°C, 5% CO_2_. The adherent cells were treated with different concentrations of PBox compounds (0–250 μM) for another 24 h. After incubation, resazurin dye was added to each well, and the plate was incubated for 6–8 h at 37°C, 5% CO_2._ Fluorescence was measured (excitation wavelength = 550 nm and emission wavelength = 590 nm) by using a multimode reader (Enspire, Perkin Elmer). The results were expressed as percent cell viability, compared with untreated cells. All assays were performed thrice in triplicates.

### Structural similarity measurements

Atom Pair fingerprints (APfp) were used for calculating the structural similarity between the PBox compounds showing good antibacterial activity (Bajusz et al., 2015; O'Boyle and Sayle, 2016). The APfp of the 13 PBox compounds and vancomycin was calculated using the sdf files downloaded from the ChemSpider for each compound. The sdf files were there loaded into the ChemMine tools software for calculating APfp of all compounds (Backman et al., 2011). The APfp were submitted to ChemmineR software for hierarchical clustering analysis (Cao et al., 2008). The Tanimoto coefficient (Tc) method was used for the cluster analysis and drawing a phylogenetic tree for calculating the relationship between the compounds.

## Results and discussion

The rate at which *S. aureus* has developed antibiotic resistance has raised serious concern for the public health. Further, biofilm infections are more dreadful and challenging to treat. An estimated 10 million deaths due to antimicrobial resistance are predicted by 2050 (Review on Antimicrobial Resistance, 2016) (https://amr-review.org/sites/default/files/160518_Final%20paper_with%20cover.pdf). The unregulated use of antibiotic has resulted in the emergence of “Superbug” such as MRSA which have developed resistance against major antibiotics and become extremely difficult to treat. Therefore, it is crucial to identify new treatment options to tackle resistant bacteria. The PBox by MMV has provided us with the platform to screen approximately 400 compounds and move a step ahead in identifying new treatment options.

**Figure 1:**
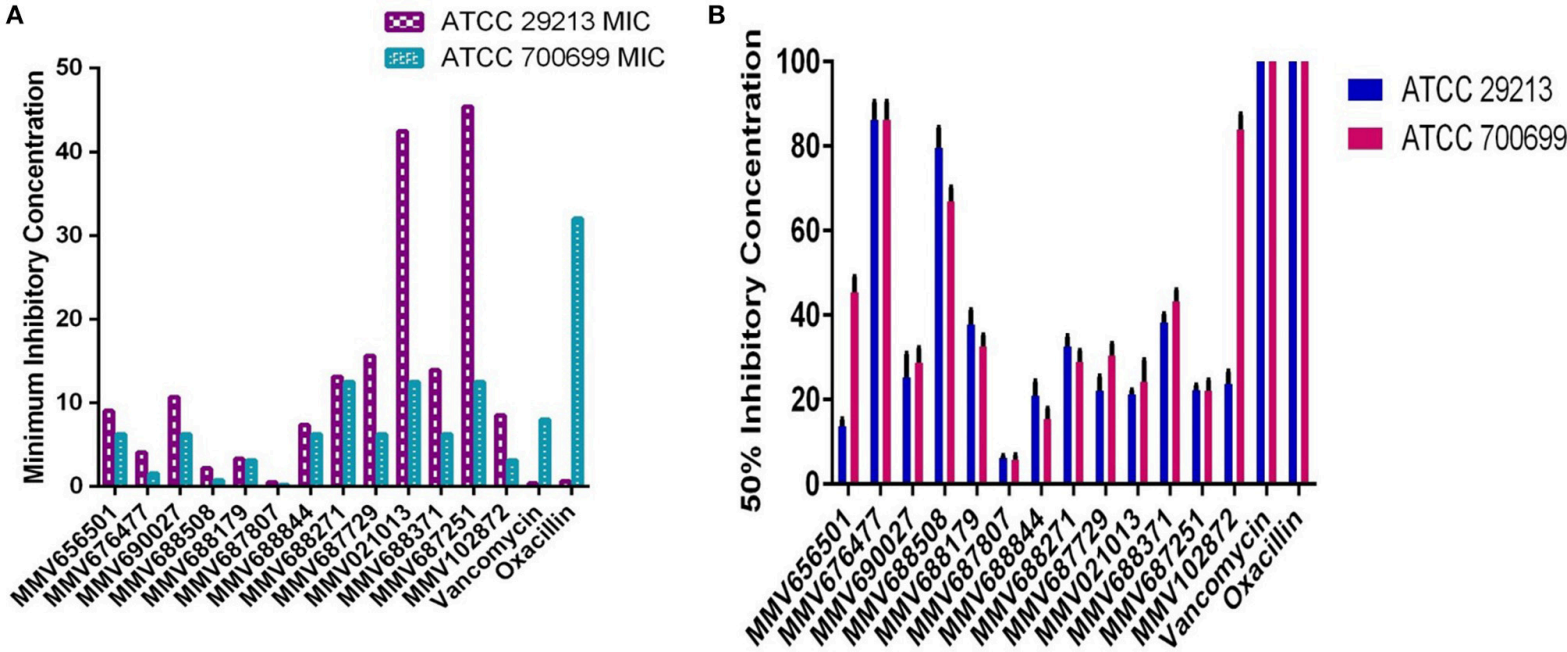
Anti-staphylococcal and anti-biofilm profiling of the 13 PBox compounds. **(A)** Anti-staphylococcal activity was determined by micro-broth dilution assay using ATCC 29213 and ATCC 700699. The bar represents the minimum inhibitory concentration (MIC). **(B)** Anti-biofilm activity was assessed by crystal violet assay against 13 PBox compounds using ATCC 29213 and ATCC 700699. The bar represents mean 50% biofilm reduction concentration ± SD.

We have screened antibacterial activity of the PBox compounds against methicillin sensitive and resistant *S. aureus* planktonic cells using broth microdilution assay (Mahato et al., 2017). Vancomycin and oxacillin were included as the internal controls in every plate. All the compounds were screened using ATCC 29213 and ATCC 700699. Out of ~400 compounds, we found 13 compounds (Figure 1, Table 1, Supplementary Table 1) with a minimum inhibitory concentration of ≤ 45 μM. These 13 compounds were grouped under Tuberculosis (*n* = 9) and Kinetoplastids (n = 4) drugs in the PBox information sheet provided by MMV. Five compounds MMV676477, MMV688179, MMV102872, MMV688508, and MMV687807, showed excellent activity against both methicillin sensitive and resistant *S. aureus* with a MIC value of ≤ 8.50 μM. Excepting MMV 688179 all the other four compounds belonged to tuberculosis disease set. Therefore, screening of the PBox revealed new compounds with anti-staphylococcal activity which were not reported earlier. Similarly, the free availability of the PBox compounds to researchers has also resulted in the identification of compounds like MMV688271, MMV688768, MMV687273 and MMV687807 showing an excellent activity against fungal pathogens (Vila and Lopez-Ribot, 2016; Mayer and Kronstad, 2017). More research into the PBox compounds also revealed compounds with anti-plasmodium activity which were not initially reported in PBox and were listed under tuberculosis or schistosomiasis (Duffy et al., 2017).

**Table 1:** Anti-staphylococcal, anti-biofilm and cytotoxicity of the 13 PBox Compounds.

**S. No**	**Compound ID**	**MMV Disease set**	**MIC (**μ**M)**		**Biofilm mass of 50% (**μ**M)**		**Cytotoxicity (IC_50_ μM)**
			**ATCC 29213**	**ATCC 700699**	ATCC 29213 ^*^	ATCC 700699^*^	
1	MMV656501	Tuberculosis	9.02	18.05	13.81 ± 1.45	45.46 ± 3.56	>250
2	MMV676477	Tuberculosis	4.06	4.06	98.44 ± 6.44	86.24 ± 4.23	>250
3	MMV690027	Kinetoplastids	10.68	10.68	25.26 ± 5.56	28.68 ± 3.45	109.32 ± 2.45
4	MMV688508	Tuberculosis	2.18	2.18	79.53 ± 4.84	66.89 ± 3.23	>250
5	MMV688179	Kinetoplastids	3.28	6.56	37.69 ± 3.49	32.54 ± 2.69	152 ± 5.41
6	MMV687807	Tuberculosis	0.50	0.50	6.20 ± 0.54	5.73 ± 1.10	>250
7	MMV688844	Tuberculosis	7.35	14.71	20.90 ± 3.40	15.43 ± 2.45	>250
8	MMV688271	Kinetoplastids	13.11	26.21	32.50 ± 2.49	28.91 ± 2.67	126.94 ± 5.52
9	MMV687729	Tuberculosis	15.61	15.61	22.15 ± 3.31	30.34 ± 2.87	169.32 ± 12.37
10	MMV021013	Tuberculosis	42.46	42.46	21.23 ± 0.98	24.27 ± 4.98	>250
11	MMV688371	Kinetoplastids	13.89	13.89	38.24 ± 1.94	43.22 ± 2.65	97.27 ± 3.07
12	MMV687251	Tuberculosis	45.41	45.41	22.23 ± 1.18	22.08 ± 2.48	220.99 ± 3.77
13	MMV102872	Tuberculosis	8.50	8.50	23.74 ± 2.90	83.98 ± 3.45	>250
14	Vancomycin	Internal Control	0.35	5.52	>100	>100	110 ± 4.66
15	Oxacillin	Internal Control	0.63	> 100	>100	>100	>250

*Vancomycin and oxacillin were used as internal controls*.

* *Significant difference was observed in the antibiofilm activity among each MMV compound and the controls (Vancomycin and Oxacillin) with p < 0.05*.

Biofilm infections are more challenging to treat due to increased tolerance to antibiotics and host defense mechanism. Therefore, it is crucial to investigate the anti-biofilm activity of the compounds showing anti-staphylococcal activity for a better treatment option in the future. Hence, the anti-biofilm activity of the 13 compounds was determined by using crystal violet (CV) assay as described previously, using ATCC 29213 and 700699. Out of the 13 compounds tested 6 compounds showed a 50% reduction in biofilm mass at ≤ 30 μM (Figure 1, Table 1). Overall, five compounds (MMV656501, MMV687807, MMV688844, MMV687729, and MMV102872) showed good activity against both planktonic and biofilm cells. MMV687807 showed excellent activity against planktonic cells with a MIC of 0.5 μM and anti-biofilm activity with an IC_50_ value of 6. 20 ± 0.54 μM and 5.73 ± 1.10 μM for methicillin-sensitive and resistant isolate respectively. However, the same compound showed a weaker inhibition of biofilm formed by *Candida albicans* (Mayer and Kronstad, 2017). Interestingly, there were two compounds (MMV021013 and MMV 687251) which were more active against biofilm as compared to planktonic cells.

**Figure 2:**
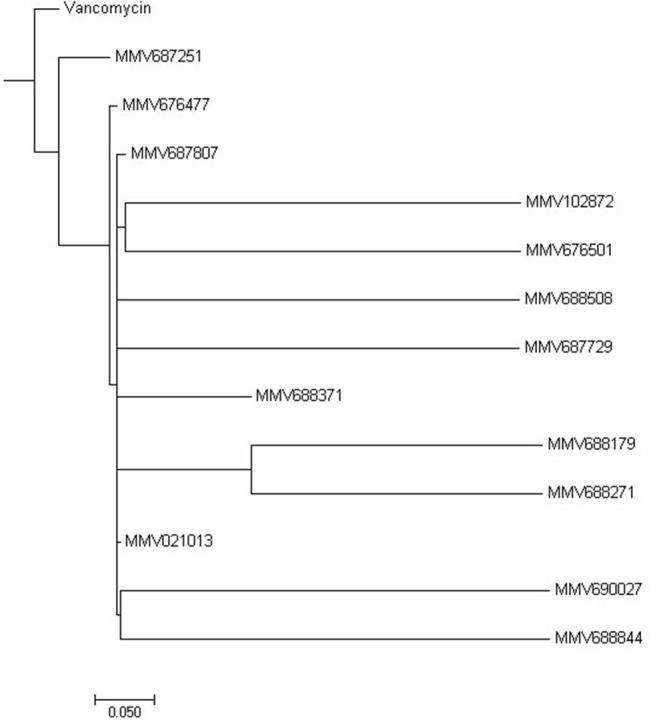
Hierarchical clustering analysis. The Hierarchical clustering analysis was performed using ChemmineR software which highlights the structural similarities between the 13 PBox compounds and vancomycin concerning their Tanimoto coefficients (Tc).

Cytotoxicity assay using RAW 264.7 mouse macrophage cell line showed all compounds to be less cytotoxic than vancomycin except MMV688371 which has comparable cytotoxicity (Table 1). The low cytotoxicity levels of these compounds reveal good potential to be taken up for *in vivo* testing in animal models in future. Additionally, to understand whether the 13 compounds shared any structural similarity, the ChemMine tools software was used, and the reference drug vancomycin was also included. The hierarchical clustering analysis (HCA) using the T_C_ method showed levels of similarities between the identified compounds ranging from 0 to 1 with higher values corresponding to greater similarities. The compounds having the T_C_ values of 1 did not imply identical compounds, however, can be predicted to have similar structural fingerprints. The HCA analysis resulted in the formation of 5 major clusters with vancomycin showing maximum structural similarity with the compounds MMV687251 and MMV676477 with T_C_ values of 0.993 and 0.960 respectively (Figure 2). The subsequent nearest clusters consist of compounds MMV687807, MMV102872 and MMV676501 (II cluster), MMV688508 and MMV687729 (III Cluster), MMV688371, MMV688179 and MMV688271 (IV Cluster) and MMV021013, MMV690027 and MMV68844 in cluster V based on the analysis. Compounds falling under different clusters indicate structural dissimilarity among them and in comparison, with vancomycin indicating a probable different antibacterial mechanism.

Therefore, our study has highlighted the identification of PBox compounds with bactericidal activity against both planktonic and biofilm cells of *S. aureus*. These compounds have the potential to be developed as a treatment option for biofilm infections, against which vancomycin also has reduced efficacy (LaPlante and Mermel, 2009).

## Conclusion

The screening of the PBox compounds has opened avenues resulting in the identification of new compounds with antimicrobial activity. These preliminary studies will help us to prioritize the compounds, to be studied at a greater depth for establishing them as a new treatment option for staphylococcal infections.

## Author contributions

VB conceived and designed the experiments. VB, SC, and UB performed the experiments. VB and PS analyzed the data. PS and VB contributed reagents, materials, and analysis tools. PS and VB wrote the paper.

### Conflict of interest statement

The authors declare that the research was conducted in the absence of any commercial or financial relationships that could be construed as a potential conflict of interest.

## Supplementary material

Supplementary Table 1 Minimum Inhibitory concentration against all MMV Pathogen box compounds.
